# Subcutaneous and Segmental Fat Loss with and without Supportive Supplements in Conjunction with a Low-Calorie High Protein Diet in Healthy Women

**DOI:** 10.1371/journal.pone.0123854

**Published:** 2015-04-15

**Authors:** Paul H. Falcone, Chih Yin Tai, Laura R. Carson, Jordan M. Joy, Matt M. Mosman, Roxanne M. Vogel, Tyler R. McCann, Kevin P. Crona, J. Daniel Griffin, Michael P. Kim, Jordan R. Moon

**Affiliations:** 1 MusclePharm Sports Science Institute, Denver, CO, United States of America; 2 Metropolitan State University of Denver, Denver, CO, United States of America; 3 University of Colorado, Boulder, CO, United States of America; 4 University of Colorado, Denver, CO, United States of America; 5 Widener University, Chester, PA, United States of America; 6 Department of Sports Exercise Science, United States Sports Academy, Daphne, AL, United States of America; Bambino Gesu' Children Hospital, ITALY

## Abstract

**Background:**

Weight loss benefits of multi-ingredient supplements in conjunction with a low-calorie, high-protein diet in young women are unknown. Therefore, the purpose of this study was to investigate the effects of a three-week low-calorie diet with and without supplementation on body composition.

**Methods:**

Thirty-seven recreationally-trained women (n = 37; age = 27.1 ± 4.2; height = 165.1 ± 6.4; weight = 68.5 ± 10.1; BMI = 25.1 ± 3.4) completed one of the following three-week interventions: no change in diet (CON); a high-protein, low-calorie diet supplemented with a thermogenic, conjugated linoleic acid (CLA), a protein gel, and a multi-vitamin (SUP); or the high-protein diet with isocaloric placebo supplements (PLA). Before and after the three-week intervention, body weight, %Fat via dual X-ray absorptiometry (DXA), segmental fat mass via DXA, %Fat via skinfolds, and skinfold thicknesses at seven sites were measured.

**Results:**

SUP and PLA significantly decreased body weight (SUP: PRE, 70.47 ± 8.01 kg to POST, 67.51 ± 8.10 kg; PLA: PRE, 67.88 ± 12.28 kg vs. POST, 66.38 ± 11.94 kg; p ≤ 0.05) with a greater (p ≤ 0.05) decrease in SUP than PLA or CON. SUP and PLA significantly decreased %Fat according to DXA (SUP: PRE, 34.98 ± 7.05% to POST, 32.99 ± 6.89%; PLA: PRE, 34.22 ± 6.36% vs. POST, 32.69 ± 5.84%; p ≤ 0.05), whereas only SUP significantly decreased %Fat according to skinfolds (SUP: PRE, 27.40 ± 4.09% to POST, 24.08 ± 4.31%; p ≤ 0.05). SUP significantly (p ≤ 0.05) decreased thicknesses at five skinfolds (chest, waist, hip, subscapular, and tricep) compared to PLA, but not at two skinfolds (axilla and thigh).

**Conclusions:**

The addition of a thermogenic, CLA, protein, and a multi-vitamin to a three-week low-calorie diet improved weight loss, total fat loss and subcutaneous fat loss, compared to diet alone.

## Introduction

Athletic, healthy women may want to reduce their body weight for personal or professional reasons, such as to achieve a specific weight class or to improve athletic performance through changes in body composition. Caloric restriction through low-calorie diets and very low-calorie diets has been used for rapid weight loss for decades, and the literature supports their effectiveness [1–5]. However, the duration of the low-calorie interventions in these studies varies from 1–3 months, and no studies have investigated rapid weight loss with an intervention lasting less than 1 month, which may be necessary in certain weight class sports. Another dietary intervention that has been gaining interest is high-protein diets. Both *ad libitum* high-protein diets [6,7] and hypocaloric high-protein diets [8–10] have induced greater weight loss than traditional high-carbohydrate, low-fat diets. However, studies with high-protein diets have also employed long intervention periods, in some cases up to 6 months [7].

In order to increase effectiveness of both short-term and long-term diets, many individuals will include weight loss supplements in their regimen. Multi-ingredient thermogenic supplements often contain either caffeine, green tea extract or both. Caffeine alone [11–13], caffeine plus tea catechins [13–16], and multi-ingredient thermogenic supplements containing caffeine [17] have been shown to induce greater energy expenditure, which could impact weight loss. Green tea extract and a multi-ingredient thermogenic supplement were demonstrated to induce loss in body weight [18,19], fat mass[19], and abdominal fat mass[20] as well. Conjugated linoleic acid (CLA) is another common weight loss supplement. Many studies have reported a decrease in body fat with CLA supplementation [21–26], and one study demonstrated reduced body weight after a 12 month CLA intervention [27]. However, the effectiveness of caffeine-containing supplements and CLA over shorter periods of time remains unclear.

Therefore, the purpose of the present study was to investigate the effectiveness of a low-calorie, high-protein diet with and without supplementation involving a thermogenic, CLA, protein, and a multi-vitamin for three weeks on body weight, total percent body fat (%Fat), segmental body fat in arms, legs and trunk, and subcutaneous body fat as measured by skinfold thicknesses at seven sites. We hypothesized that supplementation plus diet will induce lower body weight, lower total body fat, lower segmental body fat, and lower subcutaneous body fat at all seven sites over diet alone.

## Methods

### Participants

Thirty-seven women aged 18–35 volunteered to participate in this study (Table 1). Individuals were initially contacted through flyers placed in fitness clubs and nutrition stores throughout the area. Screening occurred via telephone and email. No participants had any previous medical conditions that might exclude them from the study. Exclusion criteria included any physical condition that might be contraindicated to dietary restriction, such as kidney or liver disease, heart disease, or diabetes, as reported via health history questionnaire. All participants confirmed in writing that they had no known allergies to any ingredients in the prescribed diets or supplements. Participants were included if they had a BMI between 20–35 and were all moderately active, defined by engaging in physical activity 2 or more times per week as reported via exercise status questionnaire. Exercise questionnaires indicated that subjects were all moderately active and that no significant differences existed among groups (CON: 4.1 ± 1.1 d/wk, 212.1 ± 79.3 min/wk; PLA: 4.2 ± 1.1 d/wk, 223.5 ± 97.2 min/wk; SUP 3.6 ± 1.4 d/wk, 200.1 ± 69.2 min/wk). Study procedures were approved by the MusclePharm Institutional Review Board in accordance with the Declaration of Helsinki and all participants were informed as to the possible risks of participation before written consent was given.

**Table 1:** Baseline (PRE) descriptive data and compliance of the groups.

	CON	PLA	SUP
**n**	14	13	10
**Age**	27.8 ± 3.7	26.3 ± 4.8	27.3 ± 4.5
**Height (cm)**	167.5 ± 7.3	163.9 ± 4.7	163.3 ± 6.6
**Weight (kg)**	67.6 ± 9.8	66.4 ± 11.9	67.5 ± 8.1
**BMI**	24.1 ± 3.0	25.1 ± 3.6	26.5 ± 3.6
**Dietary Compliance (%)**	100 ± 0.0	90.8 ± 7.6	88.5 ± 10

CON = control; PLA = diet + placebo; SUP = diet + supplements. No significant differences were observed between the groups for age, height, weight, BMI, and dietary compliance.

### Experimental Design

In a double-blind repeated measures design, subjects were randomly placed into one of three groups: control (CON), diet with placebo (PLA), or diet with supplementation (SUP). A low-calorie diet was followed for 3 weeks for both SUP and PLA, to which supplements or placebos were added, respectively. Subjects completed daily food and exercise logs. Before and after the dietary intervention, participants visited the laboratory for testing. Both testing sessions were identical and included: height, weight, urine specific gravity, total and segmental body fat via dual-energy X-ray absorptiometry (DXA), and 7-site skinfold.

### Pre and Post Testing Procedures

Upon arrival, subjects were asked to provide a small urine sample and urine specific gravity was measured via hand-held refractometry (Atago, Inc., Tokyo, Japan). A measurement of 1.000–1.030 was considered adequately hydrated. Height was measured to the nearest 0.1 cm on a calibrated stadiometer (Seca, Inc., Birmingham, UK). Body weight was measured to the nearest 0.01 kg with a calibrated scale. All participants wore a sports bra and athletic shorts for all body composition measurements. Skinfold subcutaneous fat thickness was measured at the following 7 sites: chest, axilla, waist, hip, thigh, subscapular, and tricep. All measurements were taken on the right side of the body by the same researcher using a Lange caliper (Beta Technology Inc., Cambridge, MD). Skinfolds were measured in duplicate to the nearest 0.5mm and averaged. %Fat was calculated using the Siri equation [28] after converting the sum of 7 skinfolds to body density using the Jackson and Pollock equation [29]. Test-retest reliability for 7-site %Fat, as measured using 14 subjects, resulted in an intraclass correlation (ICC) and a standard error of measurement (SEM) of 0.96 and 1.08%, respectively. Dual-energy X-ray absorptiometry (Lunar Prodigy Primo, General Electric, Inc., Fairfield, CT) was measured to determine %Fat, total body lean mass, and segmental fat mass, including arm, leg, and trunk. Quality assurance testing was performed daily to ensure accuracy. Test-retest reliability for DXA %Fat, as measured using 15 subjects, resulted in an ICC and SEM of >0.99 and 0.46%, respectively.

### Intervention Protocol

All participants provided a 3-day food log before the study to establish baseline values for food intake. Individuals in the CON group continued their typical diet and kept 3-day food logs weekly. The diet for the other two groups consisted of a low-calorie, high-protein diet (Bizzy Diet, MusclePharm Inc., Denver, CO), consisting primarily of meat and vegetables for 3 weeks. Since the diet involves ranges of food weight per meal (ex: 4–6 oz. of meats and 2–3 cups of vegetables for lunch and dinner), there was no specific restriction of calories that was set. The SUP group was also given protein gels (MuscleGel, MusclePharm Inc., Denver, CO; 22g of protein per serving), an encapsulated thermogenic (Burn, MusclePharm Inc., Denver, CO), a multi-vitamin (Balance, MusclePharm Inc., Denver, CO), and conjugated linoleic acid (Tone, MusclePharm Inc., Denver, CO). The PLA group was provided with an isocaloric, flavor-matched non-protein gel (maltodextrin), inert capsules, and isocaloric olive oil capsules to replace the protein gel, thermogenic, multi-vitamin, and CLA, respectively. The PLA and SUP group both kept daily food logs. All diets were carefully monitored by researchers via email and phone. Data was entered into nutrition software (Nutribase Inc., Phoenix, AZ) so that total calories, total macronutrients, and compliance could be determined. Compliance was calculated as total foods eaten that were allowed on the diet divided by total foods eaten. Nutribase uses the Atwater conversion factors to calculate calories per gram of a given food, as opposed to the 4-4-9 method. All subjects were asked to continue exercising normally.

### Statistical Analyses

A repeated measures ANOVA was used to identify any group, time and group x time interactions. When a significant interaction was found, Tukey’s *post-hoc* analyses were employed to locate where the significant differences occurred. The level of significance was set at p ≤ 0.05. Statistical analyses were performed on SPSS 14.0 (SPSS Inc. Chicago, IL). Any p-values less than 0.001 are reported as the value necessary to obtain significance (i.e. p ≤ 0.05).

## Results

### Nutritional profile

All baseline characteristics and dietary compliance are reported in Table 1. Dietary compliance was high in both diet groups (PLA: 90.8 ± 7.6%; SUP 88.5 ± 10.0%). All daily energy and macronutrient intakes are reported in Table 2. Baseline measures (PRE) of dietary intake did not differ (p > 0.05) among groups. There was a significant group x time effect (p ≤ 0.05) for total energy intake in which PLA (869 ± 157 kcal/d) and SUP (933 ± 129 kcal/d) during weeks 1–3 were significantly lower than PRE (PLA: 1610 ± 539 kcal/d; SUP: 1686 ± 331 kcal/d; p ≤ 0.05) and compared to CON (1881 ± 268 kcal/d; p ≤ 0.05). Protein intake did not differ among groups or over time; however, there was a significant group x time effect (p ≤ 0.05) for percent of calories from protein, in which PLA (38.2 ± 4.6%) and SUP (45.0 ± 5.9%) during weeks 1–3 were significantly different than PRE (PLA: 22.7 ± 10.0%; SUP: 20.8 ± 4.6%; p ≤ 0.05) and compared to CON (225.5 ± 45.5%; p ≤ 0.05). There was a significant group x time effect (p ≤ 0.05) for carbohydrate intake in which PLA (51.5 ± 13.9 g/d) and SUP (38.5 ± 12.4 g/d) during weeks 1–3 were significantly lower than PRE (PLA: 180.4 ± 86.5 g/d; SUP: 190.9 ± 29.3 g/d; p ≤ 0.05) and compared to CON (225.5 ± 45.5 g/d; p ≤ 0.05). There was a significant group x time effect (p ≤ 0.05) for percent of calories from carbohydrate in which PLA (24.0 ± 4.4%) and SUP (16.5 ± 3.9%) during weeks 1–3 were significantly lower than PRE (PLA: 42.8 ± 10.7%; SUP: 46.1 ± 8.5%; p ≤ 0.05) and compared to CON (47.7 ± 8.8%; p ≤ 0.05). Regarding fat intake, a main effect for time was observed (p = 0.006), but no group x time interaction (p > 0.05). Regarding percent of calories from fat, a main effect for time was observed (p = 0.047), but no group x time interaction (p > 0.05). There was a significant group x time effect (p = 0.014) for alcohol intake in which SUP during PRE (8.3 ± 11.7 g/d) was significantly greater than CON during PRE (4.0 ± 1.1 g/d; p = 0.005) and SUP during PRE was significantly greater than PLA during weeks 1–3 (0.9 ± 1.9 g/d; p = 0.010). There was a significant group x time effect (p = 0.043) for percent of calories from alcohol intake in which SUP during PRE (3.1 ± 3.9%) was significantly different than CON during PRE (1.8 ± 0.5%; p = 0.025) and PLA during weeks 1–3 (0.6 ± 1.3%) was significantly different from SUP during PRE (p = 0.040). Regarding fiber intake, a main effect for time was observed (p ≤ 0.05) but there was no group x time interaction (p > 0.05).

**Table 2:** Average daily energy and macronutrient intake from PRE to Weeks 1–3.

	Group	PRE	Weeks 1–3
Energy (kcal/d)**	CON	1930 ± 348	1881 ± 268
	PLA	1610 ± 539	869 ± 157^a^ ^,^ ^b^
	SUP	1686 ± 331	933 ± 129^a^ ^,^ ^b^
**Protein (g/d)**	CON	84.3 ± 34.0	80.8 ± 23.4
	PLA	88.9 ± 39.7	81.4 ± 14.9
	SUP	89.6 ± 31.9	102.3 ± 11.4
% Calories from Protein**†	CON	17.7 ± 6.8	17.0 ± 4.4
	PLA	22.7 ± 10.0	38.2 ± 4.6^a^ ^,^ ^b^
	SUP	20.8 ± 4.6	45.0 ± 5.9^a^ ^,^ ^b^
Carbohydrate (g/d)**	CON	239.6 ± 97.3	225.5 ± 45.5
	PLA	180.4 ± 86.5	51.5 ± 13.9^a^ ^,^ ^b^
	SUP	190.9 ± 29.3	38.5 ± 12.4^a^ ^,^ ^b^
% Calories from Carbohydrates**†	CON	49.4 ± 12.5	47.7 ± 8.8
	PLA	42.8 ± 10.7	24.0 ± 4.4^a^ ^,^ ^b^
	SUP	46.1 ± 8.5	16.5 ± 3.9^a^ ^,^ ^b^
Total fat (g/d)*	CON	68.8 ± 23.8	70.4 ± 18.8
	PLA	57.0 ± 27.9	35.5 ± 7.4
	SUP	57.1 ± 18.1	37.9 ±6.7
% Calories from Fat*†	CON	32.5 ± 8.9	31.7 ± 8.9
	PLA	32.3 ± 13.0	37.2 ± 3.4
	SUP	30.0 ± 5.2	37.0 ± 3.1
Total fiber (g/d)*	CON	14.8 ± 4.0	19.0 ± 6.6
	PLA	6.5 ± 1.8	7.5 ± 3.6
	SUP	7.3 ± 2.3	9.9 ± 2.1
Alcohol (g/d)**	CON	4.0 ± 1.1	6.2 ± 8.8
	PLA	4.2 ± 8.6	0.9 ± 1.9
	SUP	8.3 ± 11.7^b^ ^,^ ^c^	2.1 ± 3.2
% Calories from Alcohol**†	CON	1.8 ± 0.5	2.4 ± 3.3
	PLA	2.4 ± 4.9	0.6 ± 1.3
	SUP	3.1 ± 3.9^b^ ^,^ ^c^	1.5 ± 2.2

Representative intake was calculated as the mean of values calculated from 3-day food logs for all PRE values and CON values from weeks 1–3. Weeks 1–3 for PLA and SUP were calculated from 7-day food logs. CON = control; PLA = diet + placebo; SUP = diet + supplements.

*Main effect for time (p ≤ 0.05).

**Group-by-Time interaction (p ≤ 0.05).

†Atwater conversion factors were used to calculate calories per gram for the given foods consumed by subjects.

^a^Different from PRE (p ≤ 0.05);

^b^Different from CON (p ≤ 0.05).

^c^Different from PLA for Weeks 1–3 (p ≤ 0.05).

### Body Composition

**Fig 1:**
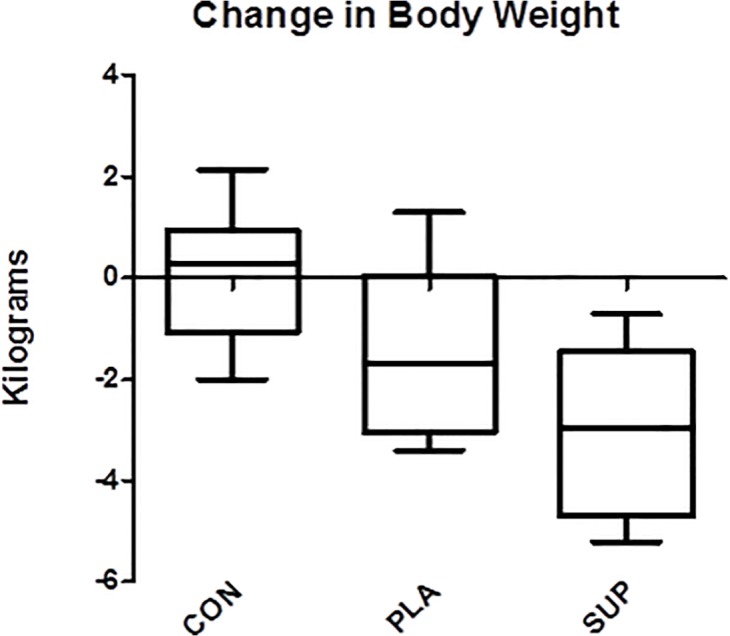
Change in body weight. Delta values for body weight from baseline to post-intervention. Solid horizontal line represents the median. Quartile 1 (Q1) and Quartile 3 (Q3) are represented by the bottom and top of the boxes respectively. The outer whiskers represent the maximum and minimum values. *Different from CON (PLA: p = 0.013; SUP: p = 0.00006). †Different from PLA (SUP: p = 0.046).

**Fig 2:**
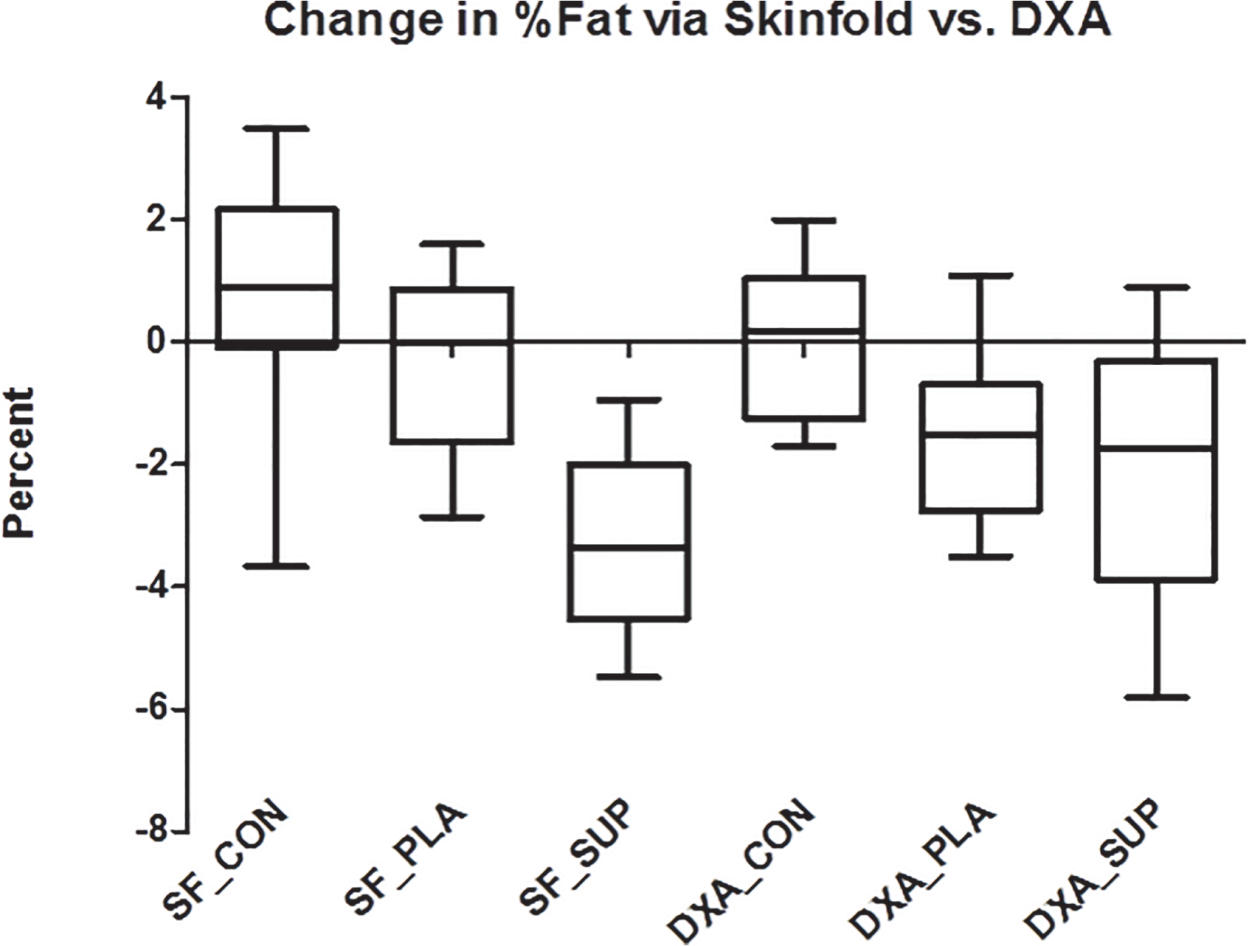
Change in %fat via skinfold vs. DXA. Delta values for %Fat from baseline to post-intervention. Solid horizontal line represents the median. Q1 and Q3 are represented by the bottom and top of the boxes respectively. The outer whiskers represent the maximum and minimum values. *Different from CON (DXA_PLA: p = 0.005; DXA_SUP: p = 0.007; SF_SUP: p = 0.00001). †Different from PLA (SF_SUP: p = 0.0001).

**Fig 3:**
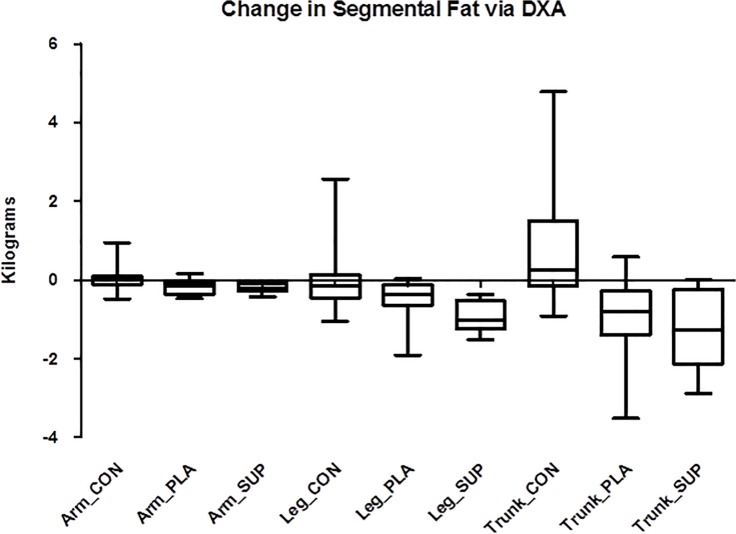
Change in segmental fat via DXA. Delta values for segmental fat from baseline to post-intervention. Solid horizontal line represents the median. Q1 and Q3 are represented by the bottom and top of the boxes respectively. The outer whiskers represent the maximum and minimum values. *Different from CON (Arm_SUP: p = 0.044; Leg_SUP: p = 0.004; Trunk_PLA: p = 0.004; Trunk_SUP: p = 0.002.)

All body weight data and body composition data measured by DXA are reported in Table 3. There was a significant group x time effect (p ≤ 0.05) for total body mass in which PLA (-1.50 ± 1.62 kg) induced greater decreases than CON (-0.01 ± 1.25 kg; p = 0.013) and SUP (-2.96 kg ± 1.66) induced greater decreases than PLA (p = 0.046) and CON (p ≤ 0.05). Individual analysis revealed that 100% of subjects in SUP (n = 10) reduced body weight (range = -0.70 to -5.2 kg) and 69.2% of subjects in PLA (n = 9) reduced body weight (range = -0.70 to -3.4 kg) (Fig 1). There was a significant group x time effect (p = 0.007) for %Fat via DXA in which SUP (-1.99 ± 2.10%) induced greater decreases than CON (0.06 ± 1.25; p = 0.007), and PLA (-1.53 ± 1.46%) induced greater decreases than CON (p = 0.005), but PLA and SUP did not differ from each other (p > 0.05). Individual analysis revealed that 80.0% of subjects in SUP (n = 14) reduced %Fat via DXA (range = -0.60 to -5.8%) and 84.6% of subjects in PLA (n = 11) reduced %Fat via DXA (range = -0.50 to -3.5%) (Fig 2). There was a significant group x time effect (p = 0.045) for segmental arm DXA, in which SUP (-0.20 ± 0.12 kg) induced greater decreases than CON (0.02 ±0.31 kg; p = 0.044)(Fig 3). There was a significant group x time effect (p = 0.005) for segmental leg DXA, in which SUP (-0.93 ± 0.41 kg) induced greater decreases than CON (0.01 ± 0.84 kg; p = 0.003). There was a significant group x time effect (p ≤ 0.05) for segmental trunk DXA, in which SUP (-1.21 ± 0.97 kg) induced greater decreases than CON (0.69 ± 1.47 kg; p = 0.002) and PLA induced greater decreases than CON (-0.90 ± 1.07 kg; p = 0.004).

**Table 3:** Changes in body composition from PRE to POST according to DXA.

DXA Results		PRE	POST	CHANGE
Body Weight**	CON	67.57 ± 9.78	67.56 ± 9.78	-0.01 ± 1.25
	PLA	67.88 ± 12.28	66.38 ± 11.94	-1.50 ± 1.62^a^ ^,^ ^b^
	SUP	70.47 ± 8.01	67.51 ± 8.10	-2.96 ± 1.66^a^ ^,^ ^b^ ^,^ ^c^
% Bodyfat**	CON	31.10 ± 7.13	31.16 ± 7.86	0.06 ± 1.25
	PLA	34.22 ± 6.36	32.69 ± 5.84	-1.53 ± 1.46^a^ ^,^ ^b^
	SUP	34.98 ± 7.05	32.99 ± 6.89	-1.99 ± 2.10^a^ ^,^ ^b^
**Lean Mass (kg)**	CON	43.32 ± 5.45	43.19 ± 5.33	-0.13 ± 0.91
	PLA	41.50 ± 7.11	41.59 ± 7.32	0.09 ± 0.84
	SUP	42.58 ± 4.05	42.21 ± 4.82	-0.36 ± 2.29
Arms FM (kg)**	CON	1.85 ± 0.77	1.87 ± 0.74	0.02 ±0.31
	PLA	2.03 ± 0.67	1.86 ± 0.58	-0.17 ± 0.20^a^
	SUP	2.13 ± 0.73	1.93 ± 0.69	-0.20 ± 0.12^a^ ^,^ ^b^
Legs FM (kg)**	CON	8.71 ± 2.70	8.72 ± 2.75	0.01 ± 0.84
	PLA	8.76 ± 2.24	8.25 ± 2.03	-0.51 ± 0.55^a^
	SUP	9.46 ± 2.66	8.54 ± 2.34	-0.93 ± 0.41^a^ ^,^ ^b^
Trunk FM (kg)**	CON	9.46 ± 3.66	10.16 ± 4.08	0.69 ± 1.47
	PLA	11.86 ± 4.66	10.96 ± 4.02	-0.90 ± 1.07^a^ ^,^ ^b^
	SUP	12.53 ± 3.87	11.32 ± 3.60	-1.21 ± 0.97^a^ ^,^ ^b^

CON = control; PLA = diet + placebo; SUP = diet + supplements.*Main effect for time (p ≤ 0.05).

**Group-by-Time interaction (p ≤ 0.05).

^a^Different from PRE (p ≤ 0.05);

^b^Different from CON (p ≤ 0.05).

^c^Different from PLA (p ≤ 0.05).

**Fig 4:**
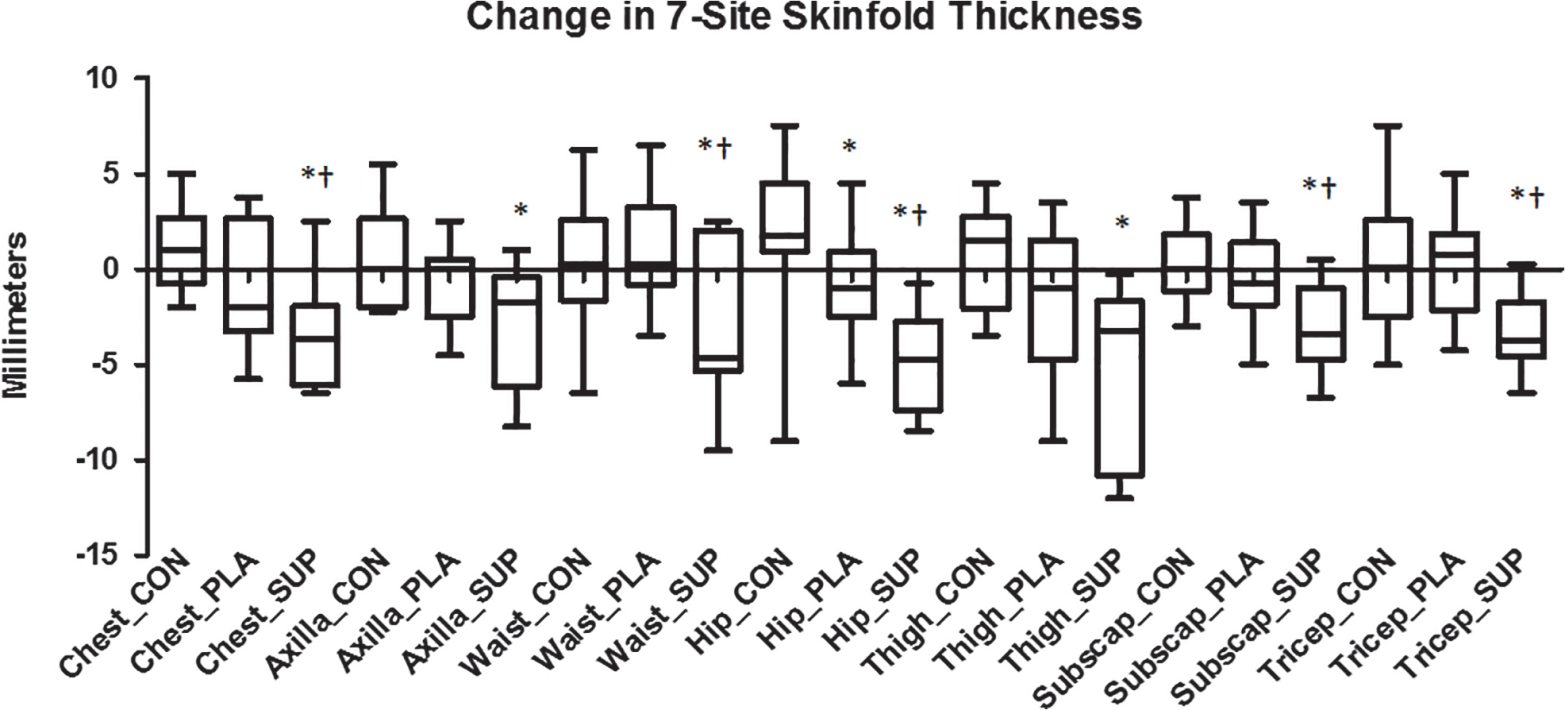
Change in 7-site skinfold thickness. Delta values for chest, axilla, waist, hip, thigh, subscapular, and tricep skinfold thicknesses from baseline to post-intervention. Solid horizontal line represents the median. Q1 and Q3 are represented by the bottom and top of the boxes respectively. The outer whiskers represent the maximum and minimum values. *Different from CON (Chest_SUP: p = 0.0003; Axilla_SUP: p = 0.006; Waist_SUP: p = 0.047; Hip_PLA: p = 0.043; Hip_SUP: p = 0.00007; Thigh_SUP: p = 0.0004; Subscapular_SUP: p = 0.001; Tricep_SUP: p = 0.006). †Different from PLA (Chest_SUP: p = 0.047; Waist_SUP: p = 0.014; Hip_SUP: p = 0.002; Subscapular_SUP: p = 0.015; Tricep_SUP: p = 0.002).

All body composition data measured by skinfolds are reported in Table 4. There was a significant group x time effect (p ≤ 0.05) for %Fat via 7-site skinfold in which SUP (-3.31 ± 1.49%) induced greater decreases than PLA (-0.36 ± 1.51%; p ≤ 0.05) and CON (0.72 ± 1.87%; p ≤ 0.05). Individual analysis revealed that 100% of subjects in SUP (n = 10) reduced %Fat via 7-site skinfold (range = -0.93 to -5.4%) and 46.2% of subjects in PLA (n = 5) reduced %Fat via 7-site skinfold (range = -0.50 to -2.8%) (Fig 2). There was a significant group x time effect (p = 0.002) for chest skinfold, in which SUP (-3.43 ± 2.92mm) induced greater decreases than PLA (-0.71 ± 3.16 mm; p = 0.047) and CON (1.07 ± 2.28 mm; p ≤ 0.05). Individual analysis revealed that 90.0% of subjects in SUP (n = 9) reduced chest skinfold (range = -0.25 to -6.3 mm) and 53.8% of subjects in PLA (n = 7) reduced chest skinfold (range = -2.0 to -5.8 mm) (Fig 4). There was a significant group x time effect (p = 0.009) for axilla skinfold, in which SUP (-2.80 ± 3.10 mm) induced greater decreases than CON (0.63 ± 2.43 mm; p = 0.006). There was a significant group x time effect (p = 0.028) for waist skinfold, in which SUP (-2.9 ± 4.04 mm) induced greater decreases than PLA (0.90 ± 2.78 mm; p = 0.014) and CON (0.30 ± 3.39 mm; p = 0.046). Individual analysis revealed that 70.0% of subjects in SUP (n = 7) reduced waist skinfold (range = -1.0 to -9.5 mm) and 46.2% of subjects in PLA (n = 6) reduced waist skinfold (range = -0.25 to -3.5 mm) (Fig 4). There was a significant group x time effect (p ≤ 0.05) for hip skinfold, in which SUP (-4.98 ± 2.71 mm) induced greater decreases than PLA (-0.81 ± 2.94 mm; p = 0.002) and CON (2.00 ± 3.89 mm; p ≤ 0.05), and PLA induced greater decreases than CON (p = 0.04). Individual analysis revealed that 100.0% of subjects in SUP (n = 10) reduced hip skinfold (range = -0.75 to -8.5 mm) and 69.2% of subjects in PLA (n = 9) reduced hip skinfold (range = -0.50 to -6.0 mm) (Fig 4). There was a significant group x time effect (p = 0.0016) for thigh skinfold, in which SUP (-5.30 ± 4.51 mm) induced greater decreases than CON (0.68 ± 2.54 mm; p ≤ 0.05). There was a significant group x time effect (p = 0.003) for subscapular skinfold, in which SUP (-3.05 ± 2.32 mm) induced greater decreases than PLA (-0.44 ± 2.35 mm; p = 0.015) and CON (0.29 ± 2.03 mm; p = 0.001). Individual analysis revealed that 90.0% of subjects in SUP (n = 9) reduced subscapular skinfold (range = -1.0 to -6.8 mm) and 53.8% of subjects in PLA (n = 7) reduced subscapular skinfold (range = -0.75 to -5.0 mm) (Fig 4). There was a significant group x time effect (p = 0.007) for tricep skinfold, in which SUP (-3.25 ± 1.97 mm) induced greater decreases than PLA (0.23 ± 2.67 mm; p = 0.015) and CON (0.45 ± 3.47 mm; p = 0.001). Individual analysis revealed that 90.0% of subjects in SUP (n = 9) reduced tricep skinfold (range = -1.0 to -6.5 mm) and 38.5% of subjects in PLA (n = 5) reduced tricep skinfold (range = -0.25 to -4.3 mm) (Fig 4).

**Table 4:** Changes in body composition from PRE to POST according to skinfolds.

Skinfold Results		PRE	POST	CHANGE
% Bodyfat**	CON	22.58 ± 2.74	23.30 ± 3.62	0.72 ± 1.87
	PLA	26.40 ± 2.93	26.04 ± 2.64	-0.36 ± 1.51
	SUP	27.40 ± 4.09	24.08 ± 4.31	-3.31 ± 1.49^a^ ^,^ ^b^ ^,^ ^c^
Chest (mm)**	CON	13.09 ± 3.07	14.16 ± 3.82	1.07 ± 2.28
	PLA	16.60 ± 3.38	15.88 ± 2.05	-0.71 ± 3.16
	SUP	18.25 ± 5.80	14.83 ± 4.60	-3.43 ± 2.92^a^ ^,^ ^b^ ^,^ ^c^
Axilla (mm)**	CON	13.30 ± 4.19	13.93 ± 3.93	0.63 ± 2.43
	PLA	16.98 ± 4.71	16.21 ± 3.97	-0.77 ± 2.04
	SUP	18.48 ± 6.70	15.68 ± 6.03	-2.80 ± 3.10^a^ ^,^ ^b^
Waist (mm)**	CON	17.89 ± 3.35	18.2 ± 4.32	0.30 ± 3.39
	PLA	23.54 ± 4.12	24.44 ± 3.54	0.90 ± 2.78
	SUP	23.40 ± 7.21	20.5 ± 5.08	-2.9 ± 4.04^a^ ^,^ ^b^ ^,^ ^c^
Hip (mm)**	CON	18.79 ± 4.74	20.82 ± 5.56	2.00 ± 3.89
	PLA	25.31 ± 5.79	24.5 ± 5.20	-0.81 ± 2.94^b^
	SUP	25.70 ± 7.02	20.73 ± 5.97	-4.98 ± 2.71^a^ ^,^ ^b^ ^,^ ^c^
Thigh (mm)**	CON	22.00 ± 2.16	22.68 ± 3.79	0.68 ± 2.54
	PLA	25.46 ± 3.78	23.79 ± 2.87	-1.67 ± 3.95
	SUP	26.25 ± 4.21	20.95 ± 3.07	-5.30 ± 4.51^a^ ^,^ ^b^
Subscapula (mm)**	CON	15.02 ± 4.07	15.3 ± 4.61	0.29 ± 2.03
	PLA	18.38 ± 6.27	17.94 ± 5.30	-0.44 ± 2.35
	SUP	21.25 ± 6.41	18.20 ± 5.53	-3.05 ± 2.32^a^ ^,^ ^b^ ^,^ ^c^
Triceps (mm)**	CON	17.02 ± 3.62	17.46 ± 4.61	0.45 ± 3.47
	PLA	20.04 ± 3.45	20.27 ± 2.74	0.23 ± 2.67
	SUP	21.13 ± 4.94	17.88 ± 4.07	-3.25 ± 1.97^a^ ^,^ ^b^ ^,^ ^c^

CON = control; PLA = diet + placebo; SUP = diet + supplements. *Main effect for time (p ≤ 0.05).

**Group-by-Time interaction (p ≤ 0.05).

^a^Different from PRE (p ≤ 0.05);

^b^Different from CON (p ≤ 0.05).

^c^Different from PLA (p ≤ 0.05).

## Discussion

In accordance with our hypothesis, the change in body weight from supplementation plus diet was significantly greater than the change in body weight from the diet alone. Though the weight loss in each group was relatively small (PLA: -1.50 ± 1.62 kg; SUP: -2.96 ± 1.66 kg), the supplements induced 97% more weight loss compared to diet alone. This is not surprising, since many studies have demonstrated that caffeine with tea catechins, which are found in the thermogenic supplement, can augment energy expenditure [13–16], and therefore weight loss. For example, Rudelle and colleagues found that energy expenditure significantly increased after consumption of 100mg of caffeine and 180mg of tea catechins 3 times per day [16], and Dulloo and associates confirmed these findings using 50mg of caffeine and 90 mg of tea catechins 3 times per day [15]. Similarly, Rumpler et al. demonstrated a similar increase in energy expenditure with oolong tea containing 54mg of caffeine and 132.5 mg of tea catechins consumed 5 times per day [13]. Caffeine alone has also been shown to increase energy expenditure [11–13]. Change in body weight in SUP and PLA were also significantly different from pre to post and compared to CON, demonstrating that the diet did induce significant weight loss. These findings are supported in the literature since low-calorie diets [30,31] have been shown to decrease body weight; however, previous studies have employed longer intervention periods, even 6 months of dieting [30]. Interestingly, the present study demonstrated that significant weight loss can occur in only 3 weeks. It is difficult to determine the main component of the diet responsible for the weight loss, since the diet was low in calories, low in carbohydrate intake, and high in protein intake. Most likely, the weight loss resulted from the combination of these factors, and future studies would be necessary to isolate the effects of each of these components. Furthermore, we analyzed total fat loss compared to caloric deficit, considering that a reduction of 3500 calories would result in one pound of fat loss. Based on the food logs, the SUP group reported a caloric deficit that should have resulted in 2.05kg lost while the PLA group should have lost 2.02kg. However, the summation of the fat measured in the arms, legs, and trunk by the DXA resulted in a 2.34kg reduction in the SUP group while the PLA group demonstrated a 1.58kg loss. Based on caloric restriction alone, the efficiency of turning a caloric deficit into fat loss in the SUP group was 114% (2.34kg/2.05kg), while the PLA group was 79% (1.58kg/2kg) effective at turning a caloric deficit into actual fat loss. These results support the hypothesis that the supportive supplements have an additive effect at reducing fat above caloric restriction alone. Specifically, the current results suggest that taking supportive supplements like those used in the current study along with following a rapid weight loss diet may enhance fat loss by 35% more (SUP: 114%—PLA: 79% = 35%) than caloric restriction alone.

In addition, supplementation plus diet induced significant reductions in skinfold thicknesses at 5 of the 7 sites measured over diet alone. However, supplementation plus diet did not have a significant effect on DXA measurements of segmental fat of the arms, legs, and trunk compared to diet alone. Though not hypothesized, these data suggest that the supplements may induce greater subcutaneous fat loss, but not total segmental fat loss. Skinfold thickness is a direct measurement of subcutaneous fat and does not account for internal fat depots. However, the DXA measurements involve the total fat of that segment, not just the subcutaneous fat. Perhaps the supplements are enabling fatty acid mobilization from subcutaneous fat depots over internal fat depots. Many compounds have induced different changes in subcutaneous and visceral depots [20,32–37] and some of these compounds are contained in the thermogenic supplement in the present study [20,34,35]. For example, Maki and colleagues demonstrated that individuals who supplemented with green tea significantly reduced their subcutaneous fat compared to control (p < 0.019), whereas intra-abdominal fat was not significantly reduced (p < 0.05) [20]. It is also possible that the skinfolds are only indicative of a small region that is being affected, whereas DXA accounts for the entire region. For example, the tricep skinfold does not include the area from the hand to the elbow, which the segmental DXA does include, and therefore a smaller effect may result from the DXA measurement due to the larger area being covered. However, this does not seem to be the case, since DXA arm fat significantly decreased in PLA over time whereas tricep skinfold thickness increased slightly. A previous study has corroborated that diet alone results in similar fat loss as measured by segmental DXA and skinfolds [30].

Similarly, SUP displayed significantly lower total body fat than PLA when calculated from skinfolds, but not when measured by DXA. Though many studies use DXA as a criterion method/gold standard for comparing other methods [38–41], this may not be accurate. Instead, some researchers choose the more accurate 4C model [42–44] or 5C model [45]. When compared to a 4C model, DXA overestimated %Fat in women at baseline and while tracking changes during weight loss [44]. In another study, both DXA and skinfold were considered precise and accurate compared to a 4C model [42]. When compared to multi-slice computed tomography (CT), DXA demonstrated high reliability [46]. If the supplements were mobilizing more fat from the subcutaneous adipose tissue, then it is possible that the skinfold calculations would be more susceptible to these changes since they rely predominately on subcutaneous fat depots over visceral fat depots. Furthermore, the skinfold equation used to predict %Fat included only skinfold thickness measurements and age so any reduction in %Fat from the skinfold equation is a direct result of reduced subcutaneous fat deposits. Thus, the %Fat as reported by the skinfold equation includes subcutaneous fat only which is why the results are not in line with DXA data. Nevertheless, the skinfold %Fat data represent changes directly related to subcutaneous adipose tissue independent of other fat and should be interpreted as such when tracking changes during a diet or exercise intervention.

One of the limitations of this study is that subjects were not followed after the intervention period to determine whether weight loss was maintained. Though subjects were educated on strategies to maintain weight loss, it was beyond the scope of this study to continue monitoring the subjects afterward. Many studies have investigated whether rapid weight loss leads to long term maintenance of weight loss, and the evidence indicates that weight regain is common [47–51]. In one study, obese individuals regained 67% of the weight lost one year following an intervention [50]. On the other hand, the same group of researchers later demonstrated a 2-fold difference in weight loss maintenance after 4 years when participants were supported with frequent treatment sessions [52]. However, these previous studies involved obese participants, whereas the focus of the present study was on the short-term efficacy of a rapid weight loss program in young, healthy, athletic women. Evidence indicates that rapid weight loss is not the best method for long-term maintenance of weight loss.

Another limitation of this study is that skinfolds are subject to observer error. However, observer error can be greatly reduced by employing the same well-trained researcher and standardized protocols [53]. Since the ICC and SEM for skinfold measurements from the same researcher were 0.96 and 1.08% respectively, the skinfold data in the present study are considered to be highly reliable. An area for future study could be to repeat this study using CT to examine intra-abdominal fat compared to subcutaneous fat, since many previous studies used this method of comparison [12,14,32,40]. Another study limitation was that multiple products with dozens of ingredients were used making it impossible to determine if any of the supplements or ingredients alone could duplicate the effects that were induced by the combination of supplements in the present study. Though previous literature would support that the thermogenic and several of the ingredients are most likely contributing to the benefits of weight loss and in particular fat loss [42,43,46], the addition of the multi-vitamin, protein, and CLA could be having an additive or synergistic effect. Therefore, the effects of each individual supplement or ingredient on body composition changes warrants further investigation. However, the purpose of the current investigation was to determine the effects of diet-induced rapid weight loss with or without typical supportive supplements.

## Conclusion

In summary, the addition of a caffeine-containing thermogenic, CLA, multi-vitamin, and protein to a low-calorie, high-protein diet for 3 weeks: 1) reduced body weight 97% more, 2) reduced %Fat according to skinfolds 9 times more, and 3) reduced skinfold thicknesses at five of the seven sites, compared to diet alone. Additionally, since the caloric deficit was similar between the SUP and PLA groups but the fat loss was different, the supplements increased the efficiency (114% vs. 79%) of converting a caloric deficit into fat loss, thereby improving fat loss over diet alone by 35%. Since skinfolds were significantly reduced with supplementation and segmental DXA measurements were unchanged, the data suggest that the supplements were affecting the selective mobilization of fatty acids from subcutaneous fat depots for oxidation over visceral and other fat depots. It is recommended that future directions in research focus on isolating the specific contributions of each supplement to the improvements in body weight and body fat. Athletic, healthy women looking to rapidly reduce body fat with a focus on subcutaneous adipose tissue should consider adding supplements such as a thermogenic, CLA, protein gels/powders, and a multi-vitamin to their nutrition intervention as results may be significantly enhanced over diet alone.
